# Design of a Remote Time-Restricted Eating and Mindfulness Intervention to Reduce Risk Factors Associated with Early-Onset Colorectal Cancer Development among Young Adults

**DOI:** 10.3390/nu16040504

**Published:** 2024-02-10

**Authors:** Manoela Lima Oliveira, Alana Biggers, Vanessa M. Oddo, Keith B. Naylor, Zhengjia Chen, Alyshia Hamm, Lacey Pezley, Beatriz Peñalver Bernabé, Kelsey Gabel, Lisa K. Sharp, Lisa Marie Tussing-Humphreys

**Affiliations:** 1 Department of Kinesiology and Nutrition, University of Illinois Chicago, Chicago, IL 60612, USA; voddo@uic.edu (V.M.O.); ahamm6@uic.edu (A.H.); kdipma2@uic.edu (K.G.); 2 University of Illinois Cancer Center, Chicago, IL 60612, USA; knaylor@uic.edu (K.B.N.);; 3 College of Medicine, University of Illinois Chicago, Chicago, IL 60612, USA; 4 Department of Biomedical Engineering, University of Illinois Chicago, Chicago, IL 60607, USA; penalver@uic.edu; 5 Institute for Health Research and Policy, University of Illinois Chicago, Chicago, IL 60612, USA

**Keywords:** time-restricted eating, mindfulness, early-onset colorectal cancer, feasibility, acceptability, stress reduction, weight management

## Abstract

Early-onset colorectal cancer (EOCRC) is defined as a diagnosis of colorectal cancer (CRC) in individuals younger than 50 years of age. While overall CRC rates in the United States (US) decreased between 2001 and 2018, EOCRC rates have increased. This research project aims to evaluate the feasibility and acceptability of Time-Restricted Eating (TRE), Mindfulness, or TRE combined with Mindfulness among young to middle-aged adults at risk of EOCRC. Forty-eight participants will be randomly assigned to one of four groups: TRE, Mindfulness, TRE and Mindfulness, or Control. Data on feasibility, adherence, and acceptability will be collected. Measures assessed at baseline and post-intervention will include body weight, body composition, dietary intake, physical activity, sleep behavior, circulating biomarkers, hair cortisol, and the gut microbiome. The effects of the intervention on the following will be examined: (1) acceptability and feasibility; (2) body weight, body composition, and adherence to TRE; (3) circulating metabolic, inflammation, and oxidative stress biomarkers; (4) intestinal inflammation; and (5) the gut microbiome. TRE, combined with Mindfulness, holds promise for stress reduction and weight management among individuals at risk of EOCRC. The results of this pilot study will inform the design and development of larger trials aimed at preventing risk factors associated with EOCRC.

## 1. Introduction

Early-onset colorectal cancer (EOCRC) is defined as a diagnosis of colorectal cancer (CRC) in individuals younger than 50 years of age. While overall CRC rates in the United States (US) decreased between 2001 and 2018, EOCRC rates increased in non-Hispanic White, Hispanic, American Indian, and Alaska Native adults but remained stable in adults identifying as non-Hispanic Black and Asian and Pacific Islander [1,2]. EOCRC is increasing more rapidly in Westernized countries, which suggests that exposure to similar risk factors common to Western culture is a contributor to EOCRC development. Approximately only 20% of new cases are attributed to genetic causes [3], which suggests that the majority of cases are attributed to modifiable risk factors rather than hereditability.

EOCRC shares many risk factors with CRC, including excess adiposity, diet quality, and microbiome dysregulation [4,5]. Obesity early in adulthood that persists with aging is strongly associated with an increased risk of EOCRC [6]. This is a concern since obesity affects 40% of young adults (20–39 years of age) in the US [7]. Obesity is associated with metabolic, hormonal, immune (i.e., insulin resistance, increased systemic inflammation, and oxidative stress), and gut microbial perturbations that can promote gene mutations that drive EOCRC tumorigenesis [8]. This suggests the importance of weight management as a preventive approach to EOCRC.

Stress may also increase the risk of EOCRC. The prevalence of perceived psychosocial stress in young adults varies widely, with studies reporting rates ranging from 20% [9]–60% [10]. Chronic psychosocial stress (CPS) adversely affects several systems of the human body, with implications for tumorigenesis. CPS can over activate the hypothalamic–pituitary–adrenal axis and sympathetic nervous system, augmenting the release of hypothalamic-pituitary-adrenal mediators such as adrenocorticotropic hormone and cortisol and sympathetic nervous system mediators such as epinephrine and norepinephrine. Cortisol can adversely impact cell cycle control and reduce DNA repair to promote carcinogenesis [11]. In response to stress, norepinephrine and epinephrine potentiate their effects on adrenergic receptors. Chronic activation of β-adrenergic receptors specifically has been implicated in carcinogenesis, with their activation affecting inflammation, proliferative signaling, genome instability, and immune evasion, which are well-established “hallmarks of cancer” [11,12,13,14]. Furthermore, studies have shown the negative effects of CPS on eating behavior (increased appetite and preference for energy-dense foods) [15], decreased insulin sensitivity [16], increased overall and central adiposity [17], increased systemic inflammation [18], and gut microbiome dysbiosis [19], resulting in an inflamed gut [20,21]—all known risk factors for colorectal carcinogenesis [22]. This suggests the importance of addressing CPS as a modifiable risk factor for the EOCRC. 

Time-Restricted Eating (TRE) may be a salient and effective approach to weight management and EOCRC prevention among individuals with obesity and CPS. TRE is a popular type of intermittent fasting that produces a ~300–500 kcal daily energy deficit by simply limiting an individual’s eating window to 4–8 h each day [23]. Individuals following TRE are asked to limit food intake to a specified time frame (e.g., 12 p.m.–8 p.m.) and no-calorie fast (i.e., only consume water or zero-calorie beverages) for the remaining hours of the day. Studies of TRE in humans show high adherence among both younger and older adults with obesity and clinically meaningful weight loss (over >5% weight loss). Moreover, TRE is associated with improvements in insulin sensitivity, systemic inflammation, oxidative stress, circulating insulin-like growth factor 1, and leptin, indicating its potential applicability for preventing EOCRC [24,25,26]. Given its simplicity, TRE may also be more acceptable for weight management among individuals with CPS compared to traditional calorie restriction given that TRE does not require individuals to count calories and continuously monitor their intake, which may serve as an additional stressor. 

Mindfulness could also play a role in cancer risk reduction. Mindfulness meditation is the practice of cultivating a moment-to-moment awareness of internal and external experience in an accepting and open manner that includes the tenets of Kabat-Zinn’s Mindfulness-Based Stress Reduction [27]. Attitudes of open inquiry, patience, suspended judgment, and compassion are encouraged and cultivated during intervention sessions. Individuals are taught to focus attention on sensations of breath, body, and objects that enter awareness, such as thoughts and emotions, with the intention to fully experience the present moment. Protocols with different forms of intervention delivery (e.g., online vs. in-person) or with protocol changes (e.g., removal of Hatha yoga) are being tested to evaluate their efficacy for reducing CPS. Mindfulness interventions have demonstrated positive effects on perceived stress [28] and on circulating cortisol concentrations [19,29]. Existing evidence also suggests that Mindfulness yields EOCRC preventive effects specific to body weight [30], insulin sensitivity [31], and systemic inflammation [32]. Small studies have tested the effects of Mindfulness on cardiovascular disease risk [33], glycemic control [34], stress reduction [35], and chronic pain management [36] with positive outcomes. However, studies have yet to prospectively investigate the effect of Mindfulness on cancer risk reduction. 

It is possible that Mindfulness interventions could reduce cancer risk in an at-risk population through multiple pathways, including physiological stress regulation, eating behavior, and immune regulation. The release of glucocorticoids (cortisol in humans and corticosterone in animals) is dysregulated in the presence of CPS. With CPS, there is a high influx of glucocorticoids, which results in prolonged activation of the hypothalamic–pituitary–adrenal axis, which may affect two established hallmarks of cancer: genome instability and tumor-promoting inflammation [11]. One meta-analysis assessed 45 articles and found a medium effect of meditation (a component of Mindfulness) in reducing circulating cortisol (through multiple different types of cortisol assessment: salivary, hair, and blood). Meditation applies to the Mindfulness scope as it is part of the standard protocol [37]. By reducing cortisol levels, Mindfulness practices likely diminish inflammation and genome instability, leading to cell mutations. 

Obesity is a modifiable risk factor for several cancers, including colorectal cancer [38]. Several studies have evaluated the effects of Mindfulness on eating behavior to support weight management. Stress-induced emotional eating is often associated with the consumption of “comfort foods” that tend to be more energy dense [39]. To determine the effect of Mindfulness on eating behavior, Fulwiler et al. used the “Three-Factor Eating Questionnaire”, an assessment that measures three aspects of eating behavior: cognitive restraint, uncontrolled eating, and emotional eating, to determine if emotional eating shifted in response to a Mindfulness intervention [40]. They presented the hypothesis that high levels of emotional eating could predict weight gain, while low levels of emotional eating could predict weight loss. They found in their study that Mindfulness was associated with reduced levels of emotional eating, but they failed to assess participant weight change [40]. A systematic review and meta-analysis conducted in 2019 evaluated 10 Mindfulness interventions on weight control and found a significant weight reduction among the Mindfulness participants when compared to control groups [41]. Therefore, Mindfulness may reduce emotional eating, energy intake, and weight gain, thus reducing the risk of colorectal cancer.

This paper describes the design of a TRE and Mindfulness intervention among young adults with obesity and CPS. Participants will be randomized to 8 weeks of TRE, Mindfulness, TRE and Mindfulness, or Control. The primary outcomes are related to feasibility and acceptability. Secondary outcomes are related to body weight, body composition, perceived stress, metabolites of the hypothalamic–pituitary–adrenal axis, circulating cardiometabolic disease risk markers, gut microbiome, and colonic inflammation. We hypothesize that the study will be feasible (i.e., recruit ≥ 50% of those approached, retain ≥ 80% of the participants, participants in the intervention groups will complete ≥ 80% of the intervention content, and all groups will complete ≥ 80% of planned assessments) and acceptable (i.e., based on a standardized acceptability survey by Pflugeisen et al. [42], that will be assessed during week 4 of the intervention and after completing the intervention). 

## 2. Materials and Methods

The experimental protocol was approved by the University of Illinois, Chicago (IRB# 2020-0498) on 18 August 2023. Participants will provide written informed consent prior to study participation. The trial is registered at ClinicalTrials.gov (NCT06022887) since 1 September 2023.

### 2.1. Study Design 

**Figure 1:**
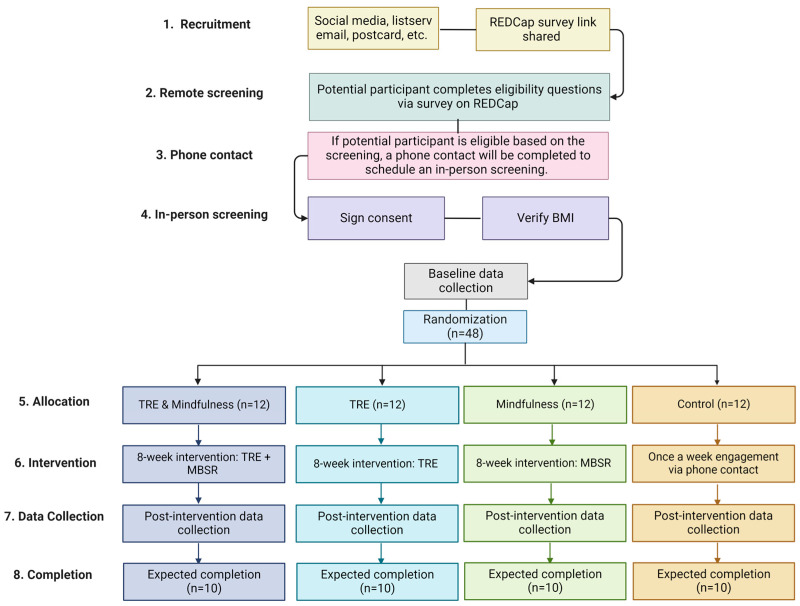
Study design. TRE = Time-restricted eating, ad libitum food intake from 12 a.m.–8 p.m.; Mindfulness: mindfulness-based stress reduction asynchronous intervention; Control = ad libitum food intake and no Mindfulness intervention (created with BioRender.com).

This study is an 8-week, parallel-arm, randomized controlled trial (Figure 1). A total of 48 young adults (18–39 years old) with obesity [body mass index (BMI) between 30–49.9 kg/m^2^] and a Perceived Stress Score ≥ 14 [43] will be randomized 1:1:1:1 to the following groups: 1. TRE; 2. Mindfulness; 3. TRE and Mindfulness; or 4. Control.

### 2.2. Setting and Recruitment

A total of 48 individuals residing in the Chicago land area will be recruited and enrolled. Recruitment will take place through social media, listserv emails, postcards, and Researchmatch.org. Interested participants will complete an online survey using a link shared through the recruitment materials. If they are found to be eligible based on the survey answers, they will be contacted via phone call and invited to come for an in-person screening. Data collection visits will take place at the University of Illinois Chicago. 

### 2.3. Participants

#### 2.3.1. Screening

Individuals interested in participating will first complete a survey to assess eligibility using a link shared via email. If an individual meets the basic inclusion criteria, they will be invited to an in-person screening to complete informed consent. They will be asked to fast for 12 h prior to their in-person screening. Individuals will be weighed using an electronic scale, and height will be assessed with a fixed stadiometer to confirm BMI (kilograms per meters squared). Individuals meeting all screening metrics (Table 1) will be invited to participate in the full trial. If the participant is eligible based on their height and weight, arrived after fasting for 12 h, and is interested in continuing in the study, baseline data collection will immediately commence. 

#### 2.3.2. Inclusion Criteria

The inclusion criteria are as follows: 18 to 39 years of age, a BMI of 30–49.99 kg/m^2^, ≥14 on the Perceived Stress Survey (PSS) [43], and owning and using a smartphone, computer, or tablet with access to the Internet.

#### 2.3.3. Exclusion Criteria

Exclusion criteria for the study includes: personal or family history of EOCRC; oral or intravenous antibiotics in the past two months; inflammatory bowel disease; genetic predisposition to colorectal cancer (e.g., Lynch syndrome); a cancer diagnosis or cancer treatment in the past 12 months; consumption of more than 50 g of ethanol daily (approximately equivalent to 4–5 12-ounce beers); combustible tobacco use; history of bariatric surgery or bowel resection; active infection; type 1 or type 2 diabetes; immunodeficiency/autoimmune disorders; using fiber or pre-/probiotic supplements > three days per week; taking corticosteroid medication (inhaled, topical, or oral) within the past two months; participating in a formal weight-loss program or those who have not maintained weight stability for three months with fluctuations—4.5 kg prior to the study; females who are pregnant or trying to become pregnant; individuals with active diagnose of any mood or eating disorders; currently following an intermittent fasting pattern or an eating window ≤ 10 h; night shift workers (shift that extends past midnight); history of eating disorder; currently taking weight loss medication; and self-reported use of illegal drugs in the past month (excluding marijuana).

#### 2.3.4. Randomization

Participants (*n* = 48) will be randomly assigned 1:1:1:1 to the following groups: (1) TRE; (2) Mindfulness; (3) TRE and Mindfulness; or (4) Control. We will use a stratified randomization approach considering sex (female vs. male) to ensure balance across treatment arms. Randomization will occur after all aspects of the baseline data collection are completed.

### 2.4. Interventions

The intervention sessions will be conducted remotely via videoconferencing (i.e., Zoom).

#### 2.4.1. Time-Restricted Eating (TRE)

Participants will be instructed to eat ad libitum from 12:00–8:00 p.m. daily and fast during the remaining hours (16-h fast). The set eating window was selected based on an observational study of 800,000 North Americans, which found that individuals usually place their TRE eating window from 12–8 p.m. so that they can have lunch and still eat dinner with friends and family [44]. During the 8-h eating window, there will be no restrictions on the types or quantities of foods consumed. In addition, participants will not be required to monitor calorie intake. During the fasting period, participants will be encouraged to drink plenty of water and consume energy-free beverages only, such as black tea or coffee. Participants will meet with a registered dietitian for 30 min at the start of the intervention to review instructions and goals and weekly thereafter. Weeks 2–8 will focus on TRE adherence and personalized problem solving related to adherence to the TRE intervention. Each day, participants will respond to a text message to indicate their eating start and stop times. If the message indicates that the participant ate only within the prescribed eating window (±30 min of 12 p.m. and + 30 min of 8 p.m.), that day will be labeled “adherent”, otherwise the day will be labeled “non-adherent.” On the same day and time each week, participants will be asked to transmit a picture of their weight from a home scale via a secure text-messaging platform. Participants will be asked to maintain their baseline level of physical activity.

#### 2.4.2. Mindfulness

Participants in this study will be granted access to the Calm.com [45] platform. Calm.com (https://www.calm.com, accessed on 22 January 2024) is a subscription-based software application to promote positive mental health [45]. Participants will have access to the “Mindfulness for Beginners” course, consisting of 30 audio lessons, each ranging from 9 to 14 min long. The title of each session is described in Table 2. During the study, participants will be asked to complete four sessions per week during weeks 1–7 and two lessons during week 8. Research staff will provide participants randomized to the Mindfulness arm with instructions on how to access the platform and a tutorial on how to navigate and engage with digital intervention. Participants will be asked to transmit a picture of their course progress via a secure text-messaging platform on the same day and time each week. 

Staff will monitor weekly usage and contact participants who engage with sessions outside of the intended protocol (i.e., less than or more than four sessions per week for weeks 1–7 of the "Mindfulness for Beginners" course) to optimize and standardize engagement. Research staff will contact participants assigned to this group weekly to track engagement in the study and answer any questions they may have. Participants will be asked to maintain their baseline level of physical activity.

#### 2.4.3. TRE and Mindfulness

This group will follow a combined protocol of the TRE and Mindfulness as described above. Participants will be asked to maintain their baseline level of physical activity.

#### 2.4.4. Control

The control group will not receive any of the interventions described. To maintain a relationship with each participant in the control group, we will contact them once a week via phone. Participants will be asked to maintain their baseline level of physical activity. At the end of the intervention period, and after all data is collected, the registered dietitian will meet with the participants for 30 min and educate them regarding the TRE protocol and provide access to the Calm.com platform. 

### 2.5. Intervention Fidelity

To assure the fidelity of the TRE intervention sessions, the content is semi-structured in a flexible format. Given the individualized nature of the sessions, the interventions cannot be fully scripted. A semi-structured approach will allow for the use of a fidelity checklist itemizing the expected general content of sessions. Non-interventionist staff (Lisa Marie Tussing-Humphreys (LTH)) will be randomly selected to observe and complete the fidelity checklist. When necessary, LTH will meet with interventionists to provide feedback from the fidelity checklist and to address deviations from the intended intervention content.

### 2.6. Data Collection and Measures

Data collection will occur at baseline and post-intervention. The following measures will be collected at both time points, unless stated otherwise:

#### 2.6.1. Body Weight and Body Composition

Body weight will be measured in a fasted state to the nearest 0.1 kg using a calibrated digital scale (Tanita BWB-800, Arlington Heights, IL, USA). Body composition will be measured by dual-energy X-ray absorptiometry (iDXA, GE Healthcare, Chicago, IL, USA) at baseline and post-intervention. Height will be measured at screening using a fixed stadiometer (Seca, Hamburg, Germany). BMI will be calculated as weight (kilograms)/height (meters squared) from screening height and measured body weights.

#### 2.6.2. Dietary Intake

We will collect two 24-h diet recalls at each time point using the Automated Self-Administered 24-h Dietary Assessment Tool (ASA-24) (US National Cancer Institute, Bethesda, MD, USA). The 24-h food recall is necessary to collect information about the participant’s diet from the previous day during a set 24-h period, 12:00 a.m. to 11:59 p.m. We will collect dietary data to explore any changes in caloric intake, diet patterns, and macro- and micronutrient intake across the study arms. At baseline, the participant will self-administer the first food recall in person during the baseline data collection. This will allow the research staff to answer any relevant questions and the participant to learn how to successfully complete this questionnaire. Participants will be asked to self-complete the second food recall 2–4 days after the baseline data collection. The third food recall will be self-completed 2–4 days prior to the participant’s post-intervention data collection. The fourth food recall will be self-completed during the post-intervention data collection visit. 

#### 2.6.3. Physical Activity and Sleep Behavior

A Fitbit accelerometer will be provided to all participants. Participants will be asked to wear the Fitbit on their wrist for seven days after the baseline data collection visit and seven days prior to the post-intervention data collection visit. The data will be processed using Fitabase (Small Steps Labs, LLC, San Diego, CA, USA) software (https://www.fitabase.com/, accessed on 22 January 2024). The purpose of accelerometry data is to determine changes in physical activity and sleep behaviors at baseline and after completing the 8-week intervention. Participants will not have to return the Fitbit throughout or after the study.

#### 2.6.4. Circulating Biomarkers 

Fasting (12 h) venous blood samples will be obtained and processed for plasma and serum; the blood will be stored at −80 °C or shipped for analysis. Glycated hemoglobin from whole blood, serum glucose, and serum insulin will be analyzed by a local commercial lab (Quest Diagnostics, Wood Dale, IL, USA). Insulin resistance will be calculated using the following homeostasis model assessment (HOMA) method: (HOMA-IR = fasting insulin [microinsulin units/milliliter] × fasting glucose [milligrams/deciliter]/405). Serum tryptophan and tryptophan metabolites (i.e., kynurenine, kynurenic acid, serotonin, and quinolinic acid) will be measured by mass spectrometry at the University of Illinois Chicago Metabolomics and Proteomics Core (Chicago, IL, USA). All samples will be measured in duplicate by a lab technician blinded to randomization assignments.

#### 2.6.5. Blood Pressure, Heart Rate, and Heart Rate Variability

Blood pressure will be assessed in duplicate with the participant in a seated position after a 10-min rest and a 5-min rest between measurements (Omron, Kyoto, Japan). Heart rate and heart rate variability will be measured for 7 days (24-h analysis) via Fitbit after the baseline data collection visit and 7 days prior to the post-intervention data collection visit. 

#### 2.6.6. Stool Collection

During the baseline visit, participants will be instructed regarding home stool collection and will receive two stool collection kits (one for baseline assessment and one for post-intervention assessment). Stools will be collected at home after the baseline visit using the kit provided by the study team. The stool kit will have a pre-labeled envelope. After collecting the sample, participants will mail the sample to the research team using a pre-labeled envelope that will be delivered within two days of shipment. The research team will provide the OMNIgene-GUT tube (DNA Genotek, Ottawa, ON, Canada) to the participants. This tube collects and stabilizes DNA for quantitative gut microbiome profile analysis and is stable at room temperature for 60 days post-collection. A Bristol stool scale will also be completed by the participant [46]. Upon receipt, stool will be aliquoted, and a portion will be processed for the calprotectin assay and stored at −80 °C until analysis. 

#### 2.6.7. Microbial Amplicon Sequencing and Bioinformatics Processing

16S rRNA amplicon sequencing will be performed using microbial DNA extracted from participant stool samples. Sample preparation and analysis will be conducted by the University of Illinois Genomics Core. The V4 region of the 16S rRNA gene will be amplified and sequenced with the Illumina MiniSeq platform (Azenta Life Sciences, Burlington, VT, USA). Environmental controls will be included in the sequences to distinguish any contaminants. Moreover, 16S rRNA reads will be processed with DADA2 (version 3.11) [47] to identify the number of counts of amplicon sequence variants (ASVs). ASVs with a low number of counts or with a low abundance (<1%) will be removed from downstream analysis. ASVs will be summarized at different phylogenetic levels and normalized using cumulative sum scaling [48]. Variation in community structure alpha (i.e., Shannon) [49] and beta diversity (e.g., Bray-Curtis distances) [50] in response to the intervention will be determined within and between groups with the vegan [51] and phyloseq [52] packages in R (R Foundation for Statistical Computing, Vienna, Austria). Compositional differences will be determined using zero-inflated generalized liner mixed models correcting for covariates (e.g., BMI, sex, age) using MaAsLin2 (version 3.18) [48,53].

#### 2.6.8. Colonic Inflammation

Fecal calprotectin is a non-invasive marker of intestinal inflammation [54]. From stool at baseline and post-intervention, fecal calprotectin will be assayed using a commercially available immunoassay kit (Bühlmann Diagnostics, Amherst, NH, USA). The lower level of detection for this assay is 32.5 μg/g. Values below the lower limit of detection are assigned a value of 16.25 μg/g per assay instructions. Values above 100 μg/g are suggestive of intestinal inflammation.

#### 2.6.9. Hair Cortisol (HCORT)

Participants will be able to decide if they are willing and able to provide hair samples (i.e., it is an opt-in measure). We will assess hair cortisol (HCORT) from a 2 cm scalp hair sample (one month’s growth represents cortisol deposition over the previous month) [55,56]. HCORT is a robust marker of the recent past (i.e., weeks or months) or an ongoing stressor that is responsive to Mindfulness [57]. Hair will be taken by the research team during the data collection appointment from the crown and vortex of the head and stored at −80 °C until analysis. Hair health will also be considered when collecting hair samples. Each hair sample will be weighed, washed twice with isopropanol, and air-dried for a minimum of 24 h. Methanol will be added to the powdered hair, and the cortisol will be extracted. Tubes are centrifuged, after which methanol extract is removed to a clean tube and evaporated using a vacuum evaporator. The dried extract is reconstituted in an assay buffer, filtered to remove particulate material, and then frozen for subsequent assays. The cortisol content of the extract is analyzed in duplicate with the control via immunoassay (Enzo Lifesciences, Farmingdale, NY, USA), which is converted to pg cortisol/mg dry hair weight, as the ELISA-based measurement of HCORT is more sensitive compared to mass spec [58]. Baseline and post-intervention samples for a participant will be assayed on the same plate.

#### 2.6.10. Adverse Event Monitoring

Intervention-related adverse events will be monitored via a standardized questionnaire and documented weekly through REDCap during the weekly check-in call. The questionnaire includes questions about gastrointestinal symptoms (nausea, vomiting, constipation, diarrhea), nervous system symptoms (dizziness, weakness, headache, fatigue, irritability, and unhappiness), any additional medication taken, COVID vaccine administration, and COVID positive status in the past week. Events will be classified according to a standardized coding scheme [59].

#### 2.6.11. Covariates That Could Influence Adherence and Intervention Effects

At baseline, participant sociodemographic status and health history will be assessed. At all in-person data collection visits, mental health (symptoms of anxiety (Generalized Anxiety Disorder-7), stress (PSS), depression (Patient Health Questionnaire-8), body image (Body Shape Questionnaire), self-efficacy, disinhibited eating (Three-Factor Eating Questionnaire), and perceived social support) [43,60,61,62,63,64,65], sleep hygiene (Pittsburgh Sleep Quality Index) [66], and medication/supplement use will be determined. 

#### 2.6.12. Power and Sample Size 

This study is designed to evaluate the feasibility of the development of a larger study. The goal of the trial is to investigate and learn about data management, data collection methodologies, and how to design a larger study in the future. Forty-eight adults will be recruited, allowing for a 20% dropout rate, resulting in completed data collection on 40 individuals, which supports the calculation of confidence intervals for feasibility and effect size components. This feasibility-pilot study is not powered to detect statistically significant effects for improvement in chronic stress and body weight or the secondary/exploratory measures. The minimum detectable difference for these outcomes is 1 standard deviation based on the sample size in each of the four groups (n = 10/arm) between the baseline and post-intervention measurements that are correlated at r = 0.5, alpha = 0.05, and 80% power. We expect that there will be no change between baseline and post-intervention measurements in the control group. We did not consider power for the overall and multiple pairwise comparisons between different groups, nor will we correct for multiple comparisons because of the exploratory nature of the pilot study [67,68,69,70,71]. 

#### 2.6.13. Data Management 

A large majority of data will be recorded directly or later uploaded into REDCap (version 14.1.3) [72], which is a secure web-based platform for building and managing online research-related databases and surveys.

### 2.7. Data Analytic Plan

Data will be managed using REDCap [72] and analyzed using the statistical software SAS (version 9.4, SAS Institute, Cary, NC, USA). The data will be organized and stored on an encrypted server. Metadata will be available and stored in an electronic lab notebook, including details of what, where, when, why, and how data were collected and processed. 

We will summarize feasibility and acceptability metrics using descriptive statistics such as frequencies, percentages, means, and standard deviations. The results will include the recruitment rate among eligible individuals approached, adherence (i.e., % interaction with asynchronous sessions, % of days adherent to the TRE protocol), % retained through post-intervention, and scores on the acceptability survey. The feasibility and acceptability rates will be estimated as the number of feasibility and acceptability participants divided by the total number of approached participants, respectively. A 95% confidence interval for the feasibility and acceptability rates will be calculated assuming a binomial distribution. Fisher’s exact test will be used to test whether feasibility and acceptability rates are significantly higher than their threshold rates (50% or 80%), respectively. A chi-square test will be used to test its relationship with other categorical clinical factors, and a *t*-test will be used for its relationship with other continuous clinical factors instead. Adjusted logistic regression (binary outcomes) or generalized linear models (GLM) (continuous outcomes) will be employed in multivariate analyses to assess their relationship with each other as clinical factors. 

Exploratory inferential statistics will be conducted, but no conclusions on the efficacy of the interventions will be drawn from the results. Initially, we will evaluate distributions, identify any outliers, and look for patterns of missing data. The continuous endpoints (stress, body weight, body composition, and cardiometabolic health) will be estimated among each of the groups, and the 95% confidence interval will be constructed assuming normal distribution, respectively. Then, a two-sample *t*-test will be utilized to compare each of the three intervention groups and the control group. GLM will be employed to estimate the adjusted difference in each of the continuous endpoints between the different groups. We will also investigate co-primary (body, weight, objective stress markers) and secondary outcomes (i.e., body composition, PSS, cardiometabolic measures) for between- and within-treatment differences and test for (treatment) × (time) interactions to compare the trends over time using generalized linear mixed models. We will investigate changes in microbial community diversity and composition (ASVs), tryptophan-related metabolites, and systemic and intestinal inflammation changes from baseline to post-intervention by treatment arm. The other binary endpoints will be compared between the different groups using Fisher’s exact test or chi-square test, respectively. Multivariate logistic regression will be further employed to compare these binary endpoints between the different groups, after adjusting for plausible confounders. Odds ratios and 95% confidence intervals will be calculated to evaluate the strength of any association that emerges.

### 2.8. Design Considerations

#### 2.8.1. Participant Retention

All participants will receive a monetary subsidy for participating in the trial. They will receive a parking voucher or USD 7 (cash) travel reimbursement for each completed study visit. If they complete the in-person screening and are not eligible to participate in the study, they will receive USD 20 (cash). If they are eligible and complete the baseline visit, they will receive USD 10 (cash) for the in-person screening and USD 60 (cash) for completing the baseline data visit (these are completed in the same visit). Once they complete the post-intervention data collection, they will receive USD 100 (cash). If they complete the study, they will receive a total of USD 170 plus transportation reimbursements. To maximize retention, we will provide financial and logistical support for transportation for the in-person data collection visits. We will also accommodate major holidays (e.g., Thanksgiving Day), allowing for an “off” day. Control participants will be provided with one free dietary weight-loss counseling session and a one-year paid membership to Calm.com after study completion.

#### 2.8.2. Participant Safety 

This trial is classified as a low-risk research study. If participants report any safety concerns, they will be addressed promptly.

#### 2.8.3. Identified A Priori Limitations

As this is a pilot study, several challenges may arise with the execution of the project. First, we may face challenges in finding participants willing to join this study. Mindfulness and TRE interventions are relatively new approaches to stress reduction and weight management that might face difficulties in acceptance. Secondly, we acknowledge the challenge of following a TRE protocol and are aware that we will require the TRE and Mindfulness group to follow the combined protocols, which could require more time commitment from the participants. This might result in differential dropout rates based on the group the participant was assigned to. Third, this study will unlikely demonstrate a significant change in EOCRC development risk, given the nature of the behavioral intervention, the small sample size, and the brief duration of the study. Lastly, the Fitbit data on heart rate variability is not validated by the U.S. Food and Drug Administration, nor are their algorithms made publicly available. However, this type of equipment may be more acceptable in a free-living environment and less biased than measurements derived from a lab environment.

#### 2.8.4. Identified A Priori Innovations

This proposal has several innovations. This will be the first study to assess Mindfulness combined with TRE among any population, including young to middle-aged adults (ages 18–39) at high risk for developing EOCRC. We focus on young adults due to EOCRC being present in adults <50 years old and it currently being on the rise in westernized countries, and we would like to focus on EOCRC prevention. This study is among the first to assess CPS both objectively [through measurement of HCORT, adrenocorticotropic hormone, epinephrine, and norepinephrine via blood] and subjectively (through application of the PSS) in the context of a combined TRE and Mindfulness intervention. HCORT is a robust measure of hypothalamic–pituitary–adrenal axis function and recent past or ongoing stress that is responsive to short-term Mindfulness interventions (<8 weeks) [73]. We will implement a remote digital Mindfulness and TRE intervention in an asynchronous format. This approach holds potential for low-cost integration into healthcare systems and for wide dissemination. Lastly, this study will attempt to explore TRE and Mindfulness’s effect on behavioral, anthropometric, hormonal, metabolic, immune, and gut microbial endpoints that link CPS to EOCRC.

## 3. Discussion and Conclusions

EOCRC is increasing in the US and other Westernized nations. Obesity and CPS are considered modifiable risk factors for the EOCRC. Our trial will be among the first to combine TRE and Mindfulness in an effort to mitigate stress and promote weight loss among young to middle-aged adults at risk of EOCRC. The feasibility and preliminary data generated here will assist in developing a fully powered efficacy trial testing the efficacy of TRE and Mindfulness for EOCRC prevention.

This study contributes to the field by assessing the combined effects of TRE and Mindfulness in a high-risk population for EOCRC. It also incorporates objective and subjective measures of CPS, explores the potential of remote digital interventions, and investigates various endpoints linking CPS to EOCRC. Findings from this study will inform the development of larger studies and provide valuable insights into stress reduction and weight management strategies for EOCRC prevention.

## Figures and Tables

**Table 1:** Inclusion and exclusion criteria.

**Inclusion Criteria**
(1) 18–39 years old.
(2) BMI: 30–49.99 kg/m^2^.
(3) Own and use a smartphone, computer, or tablet with access to the Internet.
(4) Score ≥ 14 on the Perceived Stress Score (PSS) at screening.
**Exclusion Criteria**
(1) Have a personal or family history of EOCRC.
(2) Have taken antibiotics in the previous 2 months.
(3) Have an inflammatory bowel disease or genetic predisposition to EOCRC or CRC (e.g., Lynch syndrome).
(4) Any cancer diagnosis or cancer treatment in the past 12 months.
(5) Consume >50 g of ethanol daily (approximately 4–5, 12 ounce beers).
(6) Use combustible tobacco.
(7) Have a history of bariatric surgery or bowel resection.
(8) Have an active infection.
(9) Have type 1 or type 2 diabetes, immunodeficiency/autoimmune disorder, or inflammatory bowel disease.
(10) Use fiber or pre-/probiotic supplements ≥3 days per week.
(11) Currently taking corticosteroids medication—inhaled, topical, or oral—in the past 2 months (affects cortisol measures).
(12) Are on a weight-loss diet or involved in a formal weight-loss program or are not weight stable for 3 months (+/−4.5 kg) prior to the study.
(13) Females who are pregnant or are trying to become pregnant.
(14) Have schizophrenia (medication can affect study outcomes).
(15) Have an eating window of <10 h/day or are currently following an intermittent fasting pattern.
(16) Night shift workers (shift passes midnight).
(17) Present a history of eating disorders.
(18) Currently taking weight-loss medication.
(19) Illegal drug use in the past month (not marijuana).

**Table 2:** The weekly provided Mindfulness content.

Week 1	Week 2	Week 3	Week 4	Week 5	Week 6	Week 7	Week 8
1: The Big Idea (10 min)	5: A Habit You Actually Want (10 min)	9: Into the Still point (11 min)	13: Body Wisdom (14 min)	17: The Waxy Build-up (13 min)	21: The Happiness Hit (11 min)	25: Meditation Muscle Groups (13 min)	29: Cosmic Burpee (11 min)
2: Homebase (9 min)	6: The Concentration Gym (11 min)	10: Eye of the Hurricane (10 min)	14: A Space Odyssey (13 min)	18: Welcome to the Party (10 min)	22: Strong Compassion (11 min)	26: The Do-Nothing Project (10 min)	30: Take the Power Back (13 min)
3: Pop out of your thoughts (10 min)	7: The Sweet Spot (10 min)	11: Electric Clarity (11 min)	15: Roller Coaster (13 min)	19: Slow Motion (10 min)	23: (Self) Love Bomb (13 min)	27: No Agenda (10 min)	-
4: Inner Smoothness (11 min)	8: Even Flow (10 min)	12: Sanity Day (14 min)	16: Free and Clear (14 min)	20: Better at Everything (11 min)	24: Connected from the Inside (11 min)	28: The Answer (10 min)	-

## Data Availability

No new data were created or analyzed in this study. Data sharing is not applicable to this article.
